# A comprehensive review of cluster methods for drug–drug interaction network

**DOI:** 10.1002/qub2.70015

**Published:** 2025-09-28

**Authors:** Shuyuan Cao, Guixia Liu, Xiangrun Zhou, Ji Lv

**Affiliations:** ^1^ College of Computer Science and Technology Jilin University Changchun China; ^2^ Key Laboratory of Symbolic Computation and Knowledge Engineering of Ministry of Education Jilin University Changchun China; ^3^ School of Computer Science and Technology Zhejiang Normal University Jinhua China

**Keywords:** clustering, drug combinations, drug similarity, drug‐drug interaction

## Abstract

The detection of drug–drug interaction (DDI) is crucial to the rational use of drug combinations. Experimentally, DDI detection is time‐consuming and laborious. Currently, researchers have developed a variety of computational methods to predict DDI. Although there are many reviews that summarized these computational methods, these reviews focused on supervised learning. In this review, we provide a comprehensive and systematic summary of unsupervised (i.e., clustering) methods for DDI network analysis. Unlike previous studies, we highlight the unique advantages of clustering methods DDI prediction and uncovering mechanisms of action. We first introduced common drug information and discussed how to calculate drug similarity using this drug information. Then, we introduced representative clustering algorithms (i.e., drug information‐based and network‐based methods) and described clustering evaluation metrics. Finally, we discussed the limitations and challenges in this field, and proposed potential research directions. This review aims to promote further exploration and application of clustering methods in drug combination discovery and DDI network analysis.

## 1 INTRODUCTION

Combination therapy is a promising strategy to combat antimicrobial resistance [1]. Compared to monotherapy, combination therapy (two or more drugs are used together) can enhance treatment efficacy, broaden the antimicrobial spectrum, and slow down the development of drug resistance [2, 3]. For pairwise drug combinations, there are three types: synergy effect, antagonism effect, and additive effect [4], corresponding to outcomes greater than, less than, and equal to the expected sum of individual drug effects. In microbiology laboratories, the types of drug combinations are often identified using the checkerboard assay [5]. However, this method is both time‐consuming and laborious. Therefore, it is difficult to apply for high‐throughput screening.

**FIGURE 1:**
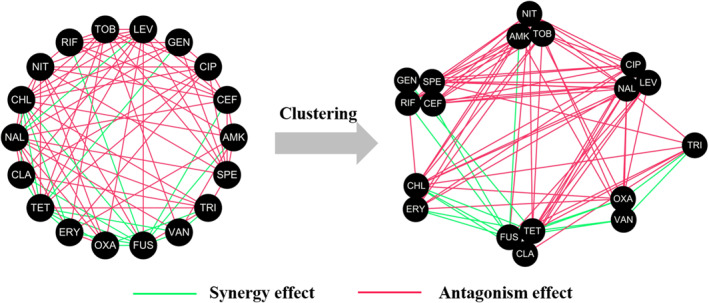
Clustering drug–drug interaction (DDI) network. Synergistic and antagonistic drug combinations are colored in green and red, respectively. In the clustered DDI networks, group–group interactions are almost monochromatic (e.g., predominantly either synergistic or antagonistic effects), and drugs within a group share similar mechanism of action.

With the accumulation of data [6, 7] and the development of computational method (e.g., machine learning, complex networks, deep learning), an increasing number computational methods have been proposed to predict drug combinations [4]. For example, Nichols et al. developed a random forest model using chemogenomics data [8] to predict drug–drug interaction (DDI) scores against *Escherichia coli* (*E. coli*) [9, 10], achieving a significant Pearson correlation coefficient of 0.52 (*p* = 10^−6^) between experimental and predicted results. However, chemogenomics data are often difficult to obtain. To overcome this limitation, computational features have been employed as alternatives. For example, Mason et al. [11] used molecular fingerprints derived from structural representations to predict drug combination types using random forest models. A molecular fingerprint is composed of a series of binary bits which are used to describe whether a particular substructure exists in the molecule [12]. The molecular fingerprint can be easily obtain using RDKit (an open‐source toolkit for cheminformatics), thus extending the scope of the model. Molecular fingerprints are interpretable and computationally efficient. However, it relies on manually designed rules and encoding methods which cannot capture complex or high‐dimensional molecular features. Deep learning can automatically learn features from molecular structures, not only retaining the local chemical environment information at the atom‐bond level [13] but also capturing long‐range interactions through attention mechanism [14], thus demonstrating higher generalization ability in tasks such as activity prediction [15] and drug combination prediction [16, 17, 18]. More details can be found in previous reviews [19, 20, 21, 22]. However, most existing reviews have focused on supervised learning and paid less attention to unsupervised learning. Unsupervised learning methods (i.e., clustering) can automatically discover potential patterns from the database (Figure 1). Specifically, clustered DDI network can be used for DDIs prediction [23], functional annotation [24, 25], and drug repurposing [26], thus providing new perspectives and methods for drug combination research.

**FIGURE 2:**
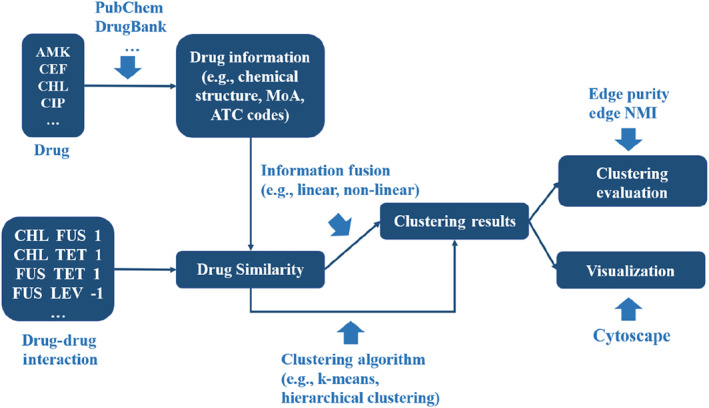
A workflow for clustering analysis of the DDI network. Drug information or DDI information can be used to calculate drug similarity. Drug similarity can be used for clustering or further integrated using linear and non‐linear methods. Clustered DDI networks can be visualized using Cytoscape. *Edge purity* and *edge NMI* can be used to assess clustering quality. ATC, anatomical therapeutic chemical; DDI, drug–drug interaction; NMI, normalized mutual information.

In this review, we systematically summarized the development and application of clustering methods for pairwise antimicrobial drug combination (Figure 2). Specifically, we first presented the data resources of drugs (e.g., chemical structure, targets, and anatomical therapeutic chemical [ATC] codes) and drug combinations. Subsequently, we introduced various methods for calculating drug similarity [27]. Then, we summarized methods for clustering DDI network and evaluation metrics. Finally, we discussed the current challenges and proposed potential research directions. This review aims to promote further application of clustering methods in drug combination research.

## 2 DATA RESOURCES FOR DRUG COMBINATIONS

In recent years, with the rapid development of high‐throughput screening technology [2, 28], a large amount of drug combination data were accumulated. As shown in Table 1, Yeh et al. [29] evaluated 210 pairwise combinations against *E. coli* MG1655, identifying 51 combinations with synergy effects. However, the types of drug combinations can vary depending on factors (e.g., bacterial strain and metabolic environment). Cokol et al. [10] systematically investigated how different metabolic conditions affect drug combination outcomes. Brochado et al. [30] assessed over 3000 pairwise combination of antibiotics, human‐targeted drugs, and food additives against 6 bacterial strains from 3 pathogens (*E. coli* BW25113 and IAI1, *Salmonella enterica serovar typhimurium* LT2 and 14,028, and *Pseudomonas aeruginosa* PAO1 and PA14). Drug combinations were dispersed across a wide range of publications, making them difficult to retrieve for further analysis. To address this problem, Lv et al. constructed an antibiotic combination database [7] and an antibiotic adjuvant combination database [6] using web crawlers and manual collection. However, the above‐mentioned databases focused on pairwise drug combinations. To handle this limitation, Tekin et al. [31] explored the patterns of higher‐order drug combination in *E*. *coli* ATCC700928.

**TABLE 1:** Drug combination datasets.

Resources	Description
Yeh et al. [29]	21 antibiotics with 210 pairwise combination against *E. coli* MG1655
Brochado et al. [30]	3000 pairwise combination of antibiotics, human‐targeted drugs, and food additives against 3 pathogens (*E. coli, Salmonella enterica serovar typhimurium, and Pseudomonas aeruginosa*)
Cokol et al. [10]	72 antibiotics with 2556 pairwise combination against *E. coli* and *Acinetobacter baumannii* in nine metabolic conditions.
Tekin et al. [31]	251 two‐drug combinations, 1512 three‐drug combinations, 5670 four‐drug combinations, and 13,608 five‐drug combinations against *E. coli* ATCC700928
ACDB [7]	304 compounds with 6175 pairwise combination against 460 bacterial strains
AADB [6]	3035 combinations of 83 antibiotics with 226 adjuvants against 325 bacterial strains

Advances in chemistry and biology provided new opportunities for mining and clustering DDI networks [8, 32, 33]. Table 2 lists the data resources available for mining DDI networks. These data resources include:

1. Drug information: 2D/3D chemical structures, SMILES, targets, physicochemical properties, ATC code, etc. SMILES is a string representation that encodes molecular structure. It enables the generation of molecular fingerprints (e.g., Morgan and MACCSS) using chemoinformatics toolkits (e.g., RDKit). These fingerprints can then be used to calculate structural similarity between drugs. Beyond structural descriptors, drug similarity can be derived from target‐based features. For example, drugs can be mapped onto the protein–protein interaction (PPI) network, and their pharmacological similarity can be quantified using network proximity [41, 42]. In addition, bacterial growth curves and ATC code can be used to calculate pharmacodynamic and therapeutic similarity, respectively.
2. Multi‐omics data: chemogenomics, proteomics, and metabolomics. In particular, functional proteomic data provide more accurate PPI [33]. For adjuvants, whose mechanism of action (MoA) are often poorly understood, non‐target metabolomics data [37, 38, 39, 43] can be used to infer potential targets. This can be achieved by comparing the metabolic profiles of drug‐treated samples with gene knockouts or knockdowns in model organisms.

**TABLE 2:** Drug datasets.

Resources	Description	References
PubChem	More than 293 million compounds and their 2D/3D chemical structures, physicochemical properties, biological activities, ATC code, etc.	[34]
DrugBank	Pharmacological data (e.g., indications, metabolic pathways, toxicity, and DDI) of 4563 FDA‐approved drugs and 6231 clinical trial drugs.	[35]
ChEMBL	More than 2 million compounds with bioactivity data (e.g., IC50, Ki, and EC50).	[36]
Mason et al.	Bacterial growth curves perturbed by different antibiotics.	[11]
Fuhrer et al.	Metabolic profiles of >3800 single gene knockouts in *E*. *coli.*	[37]
Anglada‐Girotto et al.	Metabolic profiles of >3800 single gene knockdowns (i.e., CRISPR interference) in *E*. *coli.*	[38]
Campos et al.	Metabolic profiles of 1279 drugs in *E. coli.*	[39]
STRING	Protein–protein interactions.	[40]
Nichols et al.	Chemogenomic data of *E. coli* in 324 perturbations.	[8]
Mateus et al.	Functional proteomic data of *E. coli.*	[33]

This various drug information provides a foundation for computing drug similarity and DDI network analysis.

## 3 DRUG SIMILARITY

### 3.1 Drug information‐based similarity

As we all know, an antibiotic is usually an organic compound (chemical structure) that acts on its targets, triggers perturbations in biological pathways (i.e., MoA), kills bacteria or inhibits bacterial growth (pharmacodynamics), and ultimately cures bacterial infections (clinics). Therefore, we can use these four categories of drug information to calculate drug similarity (Table 3).

**TABLE 3:** A summary of the calculation methods for drug similarity.

	Drug information	Calculation methods
Drug information‐based similarity	Molecular fingerprint	Tanimoto coefficient
	Targets	Network proximity [41]
	Bacterial growth curve	Kernel functions
	ATC code	Calculate step‐by‐step using ATC codes
Network topology‐based similarity	DDI	Cosine similarity [24]
Combined similarity	Multiple drug information	Linear integration [44], non‐linear integration [45], and network fusion [23]

#### 3.1.1 Structural similarity

To assess structural similarity, molecular representations (e.g., SMILES) are first obtained. Then, these representations are converted into molecular fingerprints (e.g., MACCS fingerprint or Morgan fingerprint) using cheminformatics tools (e.g., RDKit). Finally, the resulting binary vectors are compared using the Tanimoto coefficient in Equation (1) or the Dice coefficient in Equation (2),

(1) $$\text{Tanimoto}\left(A,B\right)=\frac{\left|A\cap B\right|}{\left|A\cup B\right|}$$

(2) $$\text{Dice}\left(A,B\right)=\frac{2\left|A\cap B\right|}{\left|A\right|+\left|B\right|}$$

where A and B are the molecular fingerprints of drug A and drug B, respectively.

The key challenge in evaluating structural similarity lies in the appropriate characterization of molecular structures. To facilitate computation, molecular structures are typically encoded into fixed‐length binary fingerprints [46]. The MACCS fingerprint is a widely used 166‐bit binary vector that encodes the presence or absence of specific predefined substructure. The Morgan fingerprint is a 2048‐bit binary vector that captures circular substructures with a defined radius (typically radius = 2). Both MACCS and Morgan fingerprints are based on 2D molecular structures and may fail to capture 3D molecular characteristics. To address this limitation, Axen et al. introduced E3FP [47], a 3D molecular fingerprint that encodes the presence of spatial molecular substructure into a 1024‐bit binary vector. MACCS, Morgan, and E3FP fingerprints are examples of bit‐fixed fingerprints. In order to encode all possible substructures, these fingerprint vectors require high dimensionality. To improve molecular representation, Duvenaud et al. proposed a convolutional neural network architecture that learns neural molecular fingerprints [13]. These representations are task‐adaptive, compact, and capable of automatically capturing relevant substructures from molecule, providing a more flexible and scalable approach to molecular representation.

#### 3.1.2 Pharmacological similarity

To evaluate pharmacological similarity between drugs, we first obtain drug targets from DrugBank [48] or previously published literature. Then, we simulate the perturbation of drugs on the PPI network using a network propagation model [42], and obtain the drug action propagating module. Finally, network proximity [41] in Equation (3) can be used to measure the interactions between DAMPs (i.e., pharmacological similarity),

(3) $$S_{\text{AB}}=\langle d_{\text{AB}}\rangle -\frac{\langle d_{\text{AA}}\rangle +\langle d_{\text{BB}}\rangle }{2}$$

where 〈*d*_AA_〉 and 〈*d*_BB_〉 are the average shortest paths of the nodes within DAMP_A_ and DAMP_B_, respectively. 〈*d*_AB_〉 is the average shortest path of nodes between DAMP_A_ and DAMP_B_, as shown in Equation (4),

(4) $$\langle d_{\text{AB}}\rangle =\frac{1}{\left|D_{A}\left|+\right|D_{B}\right|}\sum_{y\in D_{A}}\min_{x\in D_{B}}d\left(x,y\right)$$

where *D*_A_ and *D*_B_ are the DAMPs of drug A and drug B, respectively. *d*(*x*, *y*) is the shortest path between node *x* and node *y*. If *S*_AB_ < 0, then drug A and drug B are pharmacologically similar. Conversely, if *S*_AB_ ≥ 0, then drug A and drug B are pharmacologically distinct.

For adjuvants, their MoA remains unknown. In such cases, non‐target metabolomics data can be used to infer their potential targets [37, 38]. Specifically, the metabolic similarity between drug treatments and gene knockdown/knockout is calculated. Each metabolic profile is encoded as a ternary vector, as shown in Equation (5),

(5) $$Z_{A,m}=\left\{\begin{array}{cc} 1, & \text{if}\;{Z‐\text{score}}_{m}\geq {\text{thr}}_{A}\\ -1, & \text{if}\;{Z‐\text{score}}_{m}\geq {-\text{thr}}_{A}\\ 0, & \text{otherwise} \end{array}\right.$$

where *Z*_A,*m*_ is the *Z*‐score of metabolite *m* under condition A (i.e., drug treatment or gene knockout/knockdown). thr_A_ is a threshold (typically set to 2). The similarity between two ternary profiles (e.g., drug treatment and gene knockdown/knockout) is defined as Equation (6).

(6) $$S\left(Z_{\text{A}},Z_{\text{B}}\right)=\frac{Z_{\text{A}}\cdot Z_{\text{B}}}{\left|Z_{\text{A}}\left|\cdot \right|Z_{\text{B}}\right|}$$

To assess statistical significance, a hypergeometric test was performed, as shown in Equation (7),

(7) $$p_{A\;\text{versus}\;B}\left(X\geq s\right)=\sum_{k=s}^{n}\frac{\left(\begin{array}{l} K\\ k \end{array}\right)\left(\begin{array}{l} N-K\\ n-k \end{array}\right)}{\left(\begin{array}{l} N\\ n \end{array}\right)}$$

where *s* is the number of consistent changes between drug treatment and gene knockdown/knockout. *k* is the number of metabolites changed after gene knockdown/knockout. *N* is the total number of metabolites. *n* is the number of metabolites changed after drug treatment. A similar metabolic profile is typically defined by *S* ≥ 0.15 and *p* ≤ 10^−5^.

#### 3.1.3 Pharmacodynamic similarity

Under suitable conditions (e.g., a petri dish), bacteria typically proliferate following an exponential growth pattern. Upon the introduction of antibiotics, bacterial growth can be either inhibited or completely halted, depending on pharmacodynamic properties of drugs. Based on their effects on bacterial growth, antibiotics can be broadly classified into four categories: exponential‐phase bactericidal agents, lag‐phase bactericidal agents, rapid bacteriostatic agents, and slow bacteriostatic agents [49]. These pharmacodynamic categories reflect distinct killing or inhibitory dynamics. Therefore, bacterial growth curves provide valuable dynamic profiles for assessing the pharmacodynamic similarity between drugs (*S*_PD_), as shown in Equation (8),

(8) $$S_{\text{PD}}\left(A,B\right)=\exp\left(-\frac{{\left|x_{A}-x_{B}\right|}^{2}}{2\sigma^{2}}\right)$$

where ***x***_**A**_ and ***x***_**B**_ are bacterial growth curves of drug A and drug B, respectively. *σ* is a parameter.

#### 3.1.4 Therapeutic similarity

The ATC classification system [50], developed by the World Health Organization, categorizes drugs into 14 primary groups based on their anatomical, therapeutic, and chemical properties. Each drug is assigned one or more ATC codes. The ATC code consists of 7 characters including letters and numbers. The first, fourth, and fifth positions are letters, representing anatomical, therapeutic, and chemical classification; the second, third, sixth, and seventh positions are numbers, used for further subdivision. For example, erythromycin has four ATC codes (i.e., J01FA01, D1AF02, S01AA17, and D10AF52). The full breakdown of J01FA01 is: J (anti‐infective for systemic use), J01 (anti‐bacterial for systemic use), J01F (macrolides, lincosamides, and streptogramins) and J01FA (macrolides). Given this hierarchical structure, therapeutic similarity (*S*_*T*_) can be computed by comparing their ATC codes, as shown in Equation (9),

(9) $$S_{T}\left(A,B\right)=\frac{\sum_{k=1}^{3}S_{T}^{k}\left(A,B\right)}{3}$$

where $S_{T}^{k}\left(A,B\right)$ is *k*‐level therapeutic similarity, as shown in Equation (10),

(10) $$S_{T}^{k}\left(A,B\right)=\frac{\left|{\text{ATC}}_{k}\left(A\right)\cap {\text{ATC}}_{k}\left(B\right)\right|}{\left|{\text{ATC}}_{k}\left(A\right)\cup {\text{ATC}}_{k}\left(B\right)\right|},k=1,2,3$$

where ATC_*k*_(A) and ATC_*k*_(B) are *k*‐level ATC code of drug A and drug B, respectively. For drugs with multiple ATC codes, therapeutic similarity for each ATC code is calculated and then averaged.

### 3.2 Network topology‐based similarity

The above‐mentioned drug similarity is calculated using drug information. An alternative solution is to use the topological information of the DDI network [24]. In unsigned networks, node similarity is typically based on the number of shared neighbors, that is, two nodes are considered more similar if they have more common neighbors [51]. However, DDI networks are inherently signed networks, where edges carry additional semantics indicating synergistic or antagonistic effect. This complicates the notion of neighborhood and makes conventional similarity measures for unsigned networks inapplicable.

**FIGURE 3:**
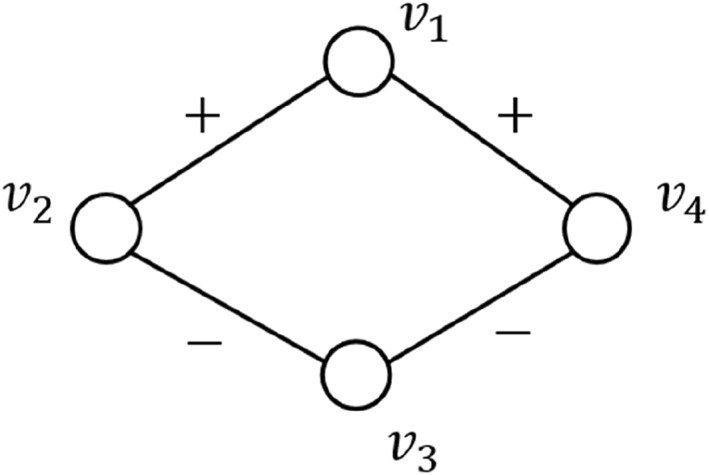
Example of a signed network.

As shown in Figure 3, $N_{1}^{+}=\left(v_{2},v_{4}\right)$, $N_{3}^{+}=\emptyset$, $N_{1}^{-}=\emptyset$, and $N_{3}^{-}=\left(v_{2},v_{4}\right)$. Although *v*_1_ and *v*_3_ share the same neighbors (i.e., *v*_2_ and *v*_4_), their *N* ^+^and *N* ^−^ are different. To address this issue, Lv et al. [24] divided common neighbors into two disjoint subsets (i.e., *S*_*c*_ and *S*_*i*_), as shown in Equations (11) and (12).

(11) $$S_{c}\left(v_{i},v_{j}\right)=\left(N_{i}^{+}\cap N_{j}^{+}\right)\cup \left(N_{i}^{-}\cap N_{j}^{-}\right)$$

(12) $$S_{i}\left(v_{i},v_{j}\right)=\left(N_{i}^{+}\cap N_{j}^{-}\right)\cup \left(N_{i}^{-}\cap N_{j}^{+}\right)$$

Therefore, the node similarity between *v*_*i*_ and *v*_*j*_ is defined as follows in Equation (13):

(13) $$S_{N}\left(i,j\right)=\frac{\left|S_{c}\left(i,j\right)\right|-|S_{i}\left(i,j\right)|}{\sqrt{\left|N_{i}\right|}\sqrt{\left|N_{j}\right|}}$$

where *N*_*i*_ and *N*_*j*_ are neighboring nodes of *v*_*i*_ and *v*_*j*_, respectively.

Alternatively, the DDI network can be represented as an adjacency matrix, as shown in Equation (14).

(14) $$A_{ij}=\left\{\begin{array}{cc} +1, & \text{synergy}\;\text{effect}\\ 0, & \text{additive}\;\text{effect}\;\\ -1, & \text{antagonism}\;\text{effect} \end{array}\right.$$

If *v*_*i*_ and *v*_*j*_ have a consistent neighbor node *v*_*k*_, then *A*_*ik*_ *A*_*kj*_ = 1; conversely, if *v*_*i*_ and *v*_*j*_ have an inconsistent neighbor node *v*_*k*_, then *A*_*ik*_ *A*_*kj*_ = −1; if *v*_*i*_ and *v*_*j*_ have no common neighbor node *v*_*k*_, then *A*_*ik*_ *A*_*kj*_ = 0. Therefore, $\left|S_{c}\left(i,j\right)\right|-\left|S_{i}\left(i,j\right)\right|=\sum_{k}A_{ik}A_{kj}$.

Because $|N_{i}|=\sum_{k}A_{ik}^{2}$ and $|N_{j}|=\sum_{k}A_{jk}^{2}$, we have the following equation:

(15) $$S_{N}\left(i,j\right)=\frac{\sum_{k}A_{ik}A_{kj}}{\sqrt{\sum_{k}A_{ik}^{2}}\sqrt{\sum_{k}A_{jk}^{2}}}$$

Equation (15) is mathematically equivalent to the cosine similarity and *S*_*N*_(*i*, *j*) ∈ [−1, 1].

### 3.3 Combined similarity

As described in previous sections, drug similarity can be computed using various drug information (e.g., chemical structure and MoA). There is both consistency and complementarity between different drug information [52]. Relying on a single data source may limit clustering performance due to incompleteness or noise. Therefore, numerous studies have explored combined similarity by integrating various drug information. These approaches can be broadly classified into three categories: linear integration [44, 53], non‐linear integration [45], and network fusion [23]. For linear integration, researchers use the geometric mean [53] or the weighted average [44] to merge multiple similarity matrices. Although simple and interpretable, linear integration may be sensitive to noise or outliers. Non‐linear integration can handle this problem; this method can integrate various drug similarities using non‐linear equations in Equation (16) [45],

(16) $$S\left(i,j\right)=1-\prod_{n}s_{ij}^{n}$$

where *n* is the number of similarity matrices, and $s_{ij}^{n}$ represents different similarity matrices. These methods are more robust to noise. In addition, we can construct networks based on different drug information, and then use a network‐based method in Equation (17) to fuse various drug information [23],

(17) $$\begin{array}{c} S_{v}^{t+1}=\sum_{k\neq v}\alpha_{k}S_{k}^{\prime }S_{v}^{t}+\left(1-\sum_{k\neq v}\alpha_{k}\right)S_{v}^{0}\\ k=1,2,3,\text{…},m \end{array}$$

where *m* is the number of similarity matrices. *α*_*k*_ is a parameter that measures the importance of each similarity matrix. $S_{k}^{\prime }=D_{k}^{-1/2}S_{k}D_{k}^{-1/2}$, where ***D***_***k***_ is a diagonal matrix.

Compared with single similarity, combined similarity enhances the robustness and completeness, particularly when drug information contain noise.

## 4 CLUSTERING DRUG‐DRUG INTERACTION NETWORK

### 4.1 Clustering method based on drug information

Drugs can be clustered based on various drug information, and then explore group‐group interactions (Table 4). For example, drugs can be categorized according to their lipophilicity into lipophilic and hydrophilic groups [57]. Yilancioglu et al. found that drug pairs with higher lipophilicity are more likely to exhibit synergistic effect [54]. Similarly, antibiotics can be classified into different categories based on their chemical structure [58], including beta‐lactams, macrolides, aminoglycosides and fluoroquinolones. The effects of combinations of aminoglycosides with beta‐lactams are most likely synergistic [59]. Another commonly used classification is based on MoA. Antibiotics can be classified into four categories: cell wall synthesis inhibitors, protein synthesis inhibitors, nucleic acid synthesis inhibitors, and folic acid synthesis inhibitors [60]. For instance, cell wall synthesis inhibitors can increase the permeability of the bacterial cell wall, facilitating the entry of protein synthesis inhibitors and thereby generating synergistic effects [61]. Furthermore, Lv et al. found that drug combinations acting on different targets of same biological pathway can bypass redundant biological mechanisms and thus produce synergistic effects [42]. According to pharmacodynamics, antibiotics can be categorized into two groups: bactericidal agents and bacteriostatic agents [62]. Ocampo et al. demonstrated that combinations of bactericidal agents with bacteriostatic agents are most likely antagonistic [55].

**TABLE 4:** A summary of the clustering method for the drug–drug interaction (DDI) network.

Method	Input	Model
Yilancioglu et al. [54]	Lipophilicity	–
Ocampo et al. [55]	Bactericidal/bacteriostatic	–
Lv et al. [23]	Chemical structure, targets, bacterial growth curve, and ATC code	Similarity matrix fusion
Yeh et al. [29]	DDI	Prism II
Guimerà et al. [56]	DDI	Stochastic block model
Lv et al. [24]	DDI	Node similarity
Lv et al. [25]	Multi‐species DDI	Similarity matrix fusion

The above‐mentioned study focuses on a specific drug information. However, each drug information has its inherent limitations. Various antibiotics have both commonalities and specificities in their chemical structures, physicochemical properties, and mechanisms of action [52]. How to integrate multi‐source drug information and obtain high‐quality clustering results is a challenge. To handle this problem, Lv et al. proposed a similarity matrix fusion algorithm [23] that integrates various drug information. Specifically, they first calculated the structural similarity, pharmacological similarity, phenotypic similarity, and therapeutic similarity, respectively. Subsequently, the four similarity matrices were fused. Finally, the fused similarity matrix was used for cluster analysis. The results showed that integrating multi‐source drug information can improve the clustering quality of the DDI network.

### 4.2 Clustering method based on network topology

Beyond drug information‐based (node information) approaches, the topological structure of the DDI network itself (edge information) provides valuable information for clustering. Several algorithms have been developed. For example, Yeh et al. developed an algorithm named Prism II [29]. The algorithm gradually merges drug groups by calculating the Euclidean distance between drug groups and monochrome entropy. The clustered DDI network is almost monochrome, and its groups are related to the known antibiotic classifications (i.e., MoA). In other words, drugs in the same group have similar mechanisms of action. One potential application is functional annotation for drugs with unknown MoA [63, 64].

Drugs can be categorized into some groups and drug–drug interactions depend on their groups. This coincides with the concept of stochastic block models. Guimerà and Sales‐Pardo proposed a stochastic block model to predict unknown drug–drug interactions [56]. The results showed that the stochastic block model is more accurate than the Prism II algorithm in most cases.

Similarly, Lv et al. proposed a method to measure drug similarity in DDI networks [24]. The similarity correlates with MoA similarity (*r* = 0.37, *p*‐value <0.01). After clustering DDI networks with the drug similarity, the clustered DDI network showed good monochromaticity, and the drug groups were highly correlated with known antibiotic classifications (i.e., MoA). However, the accuracy of the method is heavily dependent on the quality of the dataset. If there is noise in the dataset, it will have a bad impact on the clustering results. To handle this problem, Lv et al. collected and integrated multi‐species DDI [25], thus reducing the bad effect of noise on clustering. The results showed that integrating multi‐species DDI information can obtain more robust clustering results.

## 5 CLUSTERING EVALUATION

**FIGURE 4:**
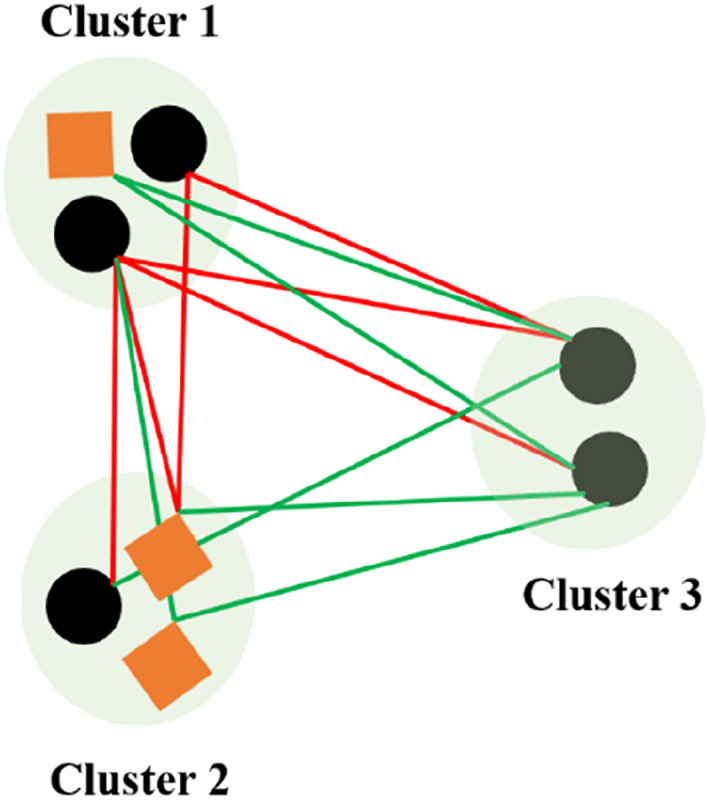
Purity and edge external evaluation criterions for cluster quality. Majority class and the number of objects of the majority class for three clusters are: circle, 2 (cluster 1); square, 2 (cluster 2); circle, 2 (cluster 3); antagonism, 3 (cluster 1–cluster 2); antagonism, 3 (cluster 1–cluster 3); and synergy, 3 (cluster 1–cluster 3). *Purity* is $(\frac{1}{8})\times \left(2+2+2\right)=0.75$. *Edge purity* is $(\frac{1}{12})\times \left(3+3+3\right)=0.75$.

The goal of the clustering is to make objects in the same group have similar characteristics. To assess the quality of clustering results, appropriate evaluation metrics are necessary. These metrics fall into main categories: node‐based index and edge‐based index (Figure 4). Node‐based index evaluates whether objects within a group share similar intrinsic properties (e.g., chemical structure and MoA), whereas edge‐based index assess the monochromaticity of network (i.e., group–group interactions are synergistic either or antagonistic).

### 5.1 Node‐based index

*purity* in Equation (18) is a simple and intuitive metric for evaluating cluster quality. It measures the proportion of correctly classified objects,

(18) $$purity\left(\Omega ,C\right)=\frac{1}{N}\sum_{k}\max_{j}\left|\omega_{k}\cap c_{j}\right|$$

where *N* is the total number of objects. Ω = (*ω*_1_, *ω*_2_, …, *ω*_*K*_) is the set of clusters and $C=\left(c_{1},c_{2},\text{…},c_{J}\right)$ is the set of classes. *ω*_*k*_ represents objects in the *k*‐th cluster. *c*_*j*_ represents objects in the *j*‐th class. *purity* ∈ [0, 1]. In the case of perfect clustering, *purity* equals 1. However, *purity* increases with the number of clusters and reaches its maximum when each object forms its own cluster. Therefore, normalized mutual information (NMI) is often used to mitigate this bias.

NMI in Equation (19) can be interpreted from the perspective of information theory,

(19) $$\text{NMI}\left(\Omega ,C\right)=\frac{I\left(\Omega ,C\right)}{\left[H\left(\Omega \right)+H\left(C\right)\right]/2}$$

where I is mutual information, as shown in Equation (20), which is used to measure the amount of information about the class when we are told what the cluster is.

(20) $$I\left(\Omega ,C\right)=\sum_{k}\sum_{j}P\left(\omega_{k}\cap c_{j}\right)\log_{2}\frac{P\left(\omega_{k}\cap c_{j}\right)}{P\left(\omega_{k}\right)P\left(c_{j}\right)}$$

where P(*ω*_*k*_), P(*c*_*j*_) and P(*ω*_*k*_ ∩ *c*_*j*_) are the probabilities of an object being in cluster *ω*_*k*_, cluster *c*_*j*_ and the intersection of *ω*_*k*_ and *c*_*j*_, respectively. H is entropy, as shown in Equation (21).

(21) $$H\left(\Omega \right)=-\sum_{k}P\left(\omega_{k}\right)\log_{2}\;P\left(\omega_{k}\right)$$

Although mutual information increases as the number of clusters increases (similar to *purity*), the normalization with extropy in Equation (19) helps correct for this.

### 5.2 Edge‐based index

While *purity* in Equation (18) and NMI in Equation (19) assess clustering from a node‐centric perspective, they do not evaluate the quality of interaction between groups (i.e., edge). To handle this problem, Lv et al. [23, 24] proposed *edge purity* and *edge NMI*, as shown in Equations (22) and (23),

(22) $$\begin{array}{c} edge\;purity=\frac{1}{N}\sum_{i}\sum_{j}\max\left(l_{ij},r_{ij}\right),\\ i=1,2,3,\text{…},k-1\\ j=i+1,i+2,\text{…},k \end{array}$$

where *N* is the number of drug combinations. *k* is the number of clusters. *l*_*ij*_ and *r*_*ij*_ are the number of synergistic and antagonistic drug combinations, respectively. Obviously, high *edge purity* means that the network is almost monochromatic. As with *purity*, *edge purity* increases with the number of clusters and reaches 1 when each drug is assigned to a separate cluster.

*edge NMI* in Equation (23) is defined as follows:

(23) $$N\text{MI}\left(L,R\right)=\frac{I\left(L,R\right)}{k}$$

where I(L,R) in Equation (24) is *edge mutual information* and is defined as follows:

(24) $$\begin{array}{c} I\left(L,R\right)=\sum_{i}\sum_{j}P\left(l^{i}\cap r^{j}\right)\;\log_{2}\;\frac{P\left(l^{i}\cap r^{j}\right)}{P\left(l^{i}\right)P\left(r^{j}\right)},\\ i\in \left(-1,1\right)\;\text{and}\;j\in \left(-1,1\right) \end{array}$$

where $L=\left(l_{12},l_{13},\text{…},l_{ij}\right)$ is the set of original group‐group interactions. $R=\left(r_{12},r_{13},\text{…},r_{ij}\right)$ is the set of clustered group–group interactions. P(*l* ^*i*^), P(*r* ^*j*^) and P(*l* ^*i*^ ∩ *r* ^*j*^) are the probabilities that the drug in *l* is *i*, the probability that the drug in *r* is *j*, and the probability that the drug in *l* is *i* and the drug in *r* is *j*, respectively. −1 and 1 indicate antagonism effect and synergy effect, respectively.

*Edge NMI* measures the amount of information that clustering knowledge increases when we are told about various types of DDI (i.e., synergistic or antagonistic). In Equation (24), if *i* = *j*, the amount of information increases. Conversely, the amount of information decreases. Perfect clustering has maximum edge mutual information. Therefore, *edge mutual information* has the same problem as *edge purity*. To handle this problem, we can divide the mutual information by the number of clusters (*k*) to obtain *edge NMI* in Equation (23).

## 6 CHALLENGES AND FUTURE PERSPECTIVES

Clustering is a promising approach for exploring DDI networks. In this review, we provided a systematic summary of clustering methods for DDI networks. Despite the notable progress, several challenges remain to be addressed.

First, most existing DDI network clustering studies focused on antibiotic‐antibiotic networks [25, 55] with limited attention given to antibiotic‐adjuvant network. Since the MoA of adjuvants are typically unknown [6], clustering based on drug information (i.e., MoA) becomes challenging. While network topology‐based methods offer an alternative, they are highly dependent on the quality of drug combination datasets [24, 25]. In practice, topology information of DDI network and some node information (e.g., MoA of antibiotics) are often available. Therefore, semi‐supervised clustering [65], which incorporates prior knowledge into the clustering process, is a very promising method for clustering antibiotic‐adjuvant network.

Second, in the era of big data, drug and drug combination data can be collected from multiple perspectives such as multi‐species DDI and diverse drug information (e.g., chemical structure and MoA) [6, 7]. These data sources exhibit both consistency and complementarity [52]. Although some fusion strategies (e.g., linear [44, 53], non‐linear [45], or network‐based [23]) have been proposed, they are not applied to the case of missing modality. Recent developments in multi‐modal learning with missing modality [66], including modality augmentation and feature space engineering, offer new opportunities to address this issue.

Third, determining the optimal number of clusters is a key issue in clustering analysis. Selecting the appropriate number of clusters is essential for ensuring the accuracy and interpretability of clustering results. In general, this is done by evaluating performance metrics (e.g., *purity*) over arrange of candidate values [23, 24, 25]. However, this brute‐force search is inefficient. To address this problem, we can model the process of determining the number of clusters as a Markov decision process and then apply reinforcement learning for automatic determination [67].

Lastly, most existing DDI network clustering studies focused on pairwise drug combinations [23, 24, 25, 55], whereas high‐order drug combinations remain underexplored. Traditional DDI networks, where nodes represent drugs and edges represent DDI, are inherently limited to modeling pairwise relationships. Yet, many FDA‐approved drug combinations involve 3–4 drugs [68]. Hypergraph offer a solution by representing high‐order interactions through hyperedges [69]. This enables more flexible modeling of synergy effect and antagonism effect involving multiple drugs [70] and opens up a new avenue for investigating patterns of high‐order DDI [31].

## AUTHOR CONTRIBUTIONS

**Shuyuan Cao**: Investigation; methodology; writing—original draft. **Guixia Liu**: Funding acquisition; supervision; writing—review and editing. **Xiangrun Zhou**: Investigation; visualization; writing—original draft. **Ji Lv**: Conceptualization; project administration; supervision; writing—original draft; writing—review and editing.

## CONFLICT OF INTEREST STATEMENT

The authors declare no conflicts of interest.

## Data Availability

This review does not report any original data or code nor were any new datasets or software created.
